# Assessing Corrosion in NdFeB Rails of a Superconducting MagLev Prototype

**DOI:** 10.1021/acsomega.5c07354

**Published:** 2025-11-04

**Authors:** Ana Laura D. M. Campista, Richard M. Stephan, Simone L. D. C. Brasil, Ladimir J. Carvalho

**Affiliations:** † School of Chemistry, Federal University of Rio de Janeiro (UFRJ), Rio de Janeiro 21941-909, Rio de Janeiro, Brazil; ‡ COPPE, Federal University of Rio de Janeiro (UFRJ), Rio de Janeiro 21941-598, Rio de Janeiro, Brazil

## Abstract

This study aims to evaluate the progression of the corrosion process in Neodymium–Iron–Boron (NdFeB) magnets that constitute the rails of the Superconducting Magnetic Levitation Vehicle. Periodic inspections were carried out on the tracks, including collecting corrosion product samples detached from the structure and rainwater samples, to determine the main constituents of the electrolyte to which the magnets are exposed. These rainwater samples were characterized by using ion chromatography, pH measurements, and conductivity analysis. In addition to sample collection, the inspections included temperature measurements of the metallic structure and a visual analysis of the rails. Based on the collected data, bench tests, electrochemical polarization, and immersion tests were conducted to simulate exposure conditions. The samples of NdFeB magnets subjected to immersion tests were analyzed using Scanning Electron Microscopy (SEM) and Energy Dispersive X-ray Spectroscopy (EDS), revealing that the corrosion process progresses gradually, forming a uniform passivating layer after 45 days of immersion. However, this layer detaches entirely after 60 days, restarting the corrosion process and leading to a loss of solid material from the structure.

## Graphical abstract

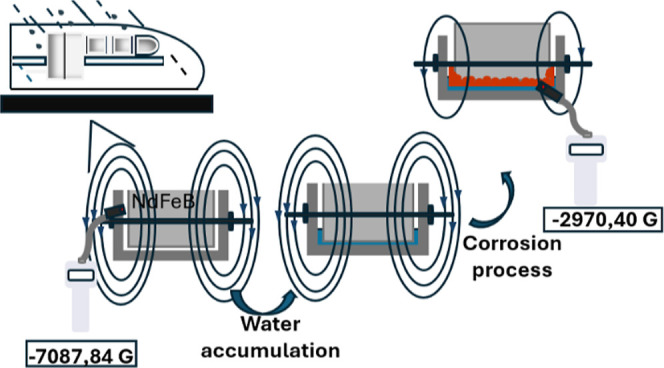

## 1 Introduction

NdFeB magnets are magnetic alloys used in various fields, including computer components, audio systems, electric vehicle components, wind turbines, and the medical industry.^1,2^ In addition to these applications, NdFeB magnets are also used as rail components in magnetic levitation vehicles, such as the MagLev Cobra, located at the Technological Center of the Federal University of Rio de Janeiro.

A key factor that directly affects the magnetic properties of these magnets is the temperature. The Curie temperature corresponds to the transition from the ferromagnetic state to the paramagnetic state. The literature indicates that NdFeB magnets exhibit a Curie temperature of approximately 312 °C.^3^

Despite the excellent magnetic performance, NdFeB magnets have low corrosion resistance.^4^ According to the literature, the susceptibility to corrosion is attributed to the microstructure, since different phases generate microgalvanic cells with multiple anodic and cathodic regions.^5,6^ The negative effects of corrosion processes on NdFeB magnets extend beyond material degradation, significantly impacting the efficiency of the generated magnetic field.^7^ The susceptibility to corrosion leads to challenges to the longevity of the NdFeB magnets, which demands understanding of the corrosion process and implementing effective prevention methods.

Given the widespread applications of NdFeB magnets, it is essential to investigate the corrosion mechanisms according to the exposed environments. Zhang et al. studied the propagation of corrosion in NdFeB magnets immersed in a 3.5 wt % NaCl solution and its effects on the material’s magnetic properties. Their findings indicated that the corrosion process begins with pitting, progresses to intergranular corrosion, and eventually leads to phase detachment.^8^

Popescu et al. examined the corrosive effects of three different electrolytes (0.5 mol·L^–1^ KOH, 0.5 mol·L^–1^ KClO_4_, and 3 wt % NaCl), reporting the highest corrosion rate in KClO_4_ solution.^9^ Additionally, Jinlin et al. assessed the corrosion resistance of NdFeB samples exposed to acidic environments (HCl, H_2_SO_4_, and HNO_3_ solutions) at concentrations ranging from 1% to 10% (w/w) for different immersion periods. Their results indicated that NdFeB exhibited lower corrosivity in HNO_3_ compared to those in the other acids. Regarding the corrosion mechanism, the authors observed that samples exposed to HCl and H_2_SO_4_ predominantly underwent intergranular corrosion, whereas those in contact with HNO_3_ exhibited uniform corrosion.^10^

The material selection, alongside the exposure environment, directly influences the corrosion process and requires careful consideration.

The various applications of NdFeB magnets expose them to different environmental conditions, making them susceptible to electrochemical reactions at the material–medium interface.

This study presents a study that focuses on applying NdFeB magnets in a magnetic levitation vehicle, the MagLev Cobra, where the rails, fixed to an outdoor platform since 2013, are subjected to temperature variations, wind, rain, and pollution, creating unique exposure conditions for the material. Therefore, this research aims to conduct a case study by assessing the operational conditions under which the MagLev Cobra rails are exposed and evaluating the progression of the corrosion process over extended outdoor exposure. Additionally, the study investigates the corrosion mechanism through immersion tests using a synthetic electrolyte.

## 2 Methodology

### 2.1 Assessment of Operational Conditions

The MagLev Cobra tracks are 200 m long where the vehicle moves back and forth between two stations. It is possible to observe in the work of Stephan et al.^11^ that the rail structure are a junction of three different materials: blocks of NdFeB magnets, some blocks of carbon steel that avoid the contact between the parts of NdFeB, and for the last, a gutter and screws of stainless steel that supports the blocks and keeps the system parts together.

The magnets were initially exposed to the environment with a factory-applied protective layer—a galvanized zinc coating—without any additional protection until the first signs of corrosion appeared. Since the zinc coating provides active protection, it was gradually consumed over time, exposing the magnets to the electrolyte and initiating the corrosion process.

Upon detecting the first signs of corrosion, the operators attempted to mitigate the process by applying two anticorrosion coatings: one based on niobium pentoxide and another zinc-based coating; the latter was applied both with and without a waterproofing membrane as a preliminary layer.

As corrosion progressed, periodic inspections along the track became necessary to assess the degree of rail degradation, the locations and extent of corrosion, and the factors contributing to the acceleration of the oxidation process. Additionally, these evaluations provided essential data to reproduce the operational conditions in laboratory experiments.

Field measurements were conducted on MagLev Cobra rails. Initially, rail temperature was measured over three months using a Medtec infrared pyrometer. Measurements were taken three times a week at three different times of the day, between 8 a.m. and 5 p.m. Rainwater samples were collected by placing collection containers along the rail platform, as shown in Figure 1. After collection, the water samples were analyzed by using ion-exchange liquid chromatography in a Metrohm ion chromatograph. In addition to the previously mentioned analyses, magnetic field measurements were conducted along the length of the track to quantify the reduction in field strength as the corrosion process progressed. These measurements were performed along already oxidized segments of the track and on recently installed rails that exhibited no apparent signs of corrosion, with the latter serving as baseline (time-zero) field reference values. The magnetic field measurements were carried out using a Gaussmeter TLMP-HALL, with readings taken in the region of the magnetic field concentrator, following the methodology described by Mello (2022).^12^

**Figure 1:**
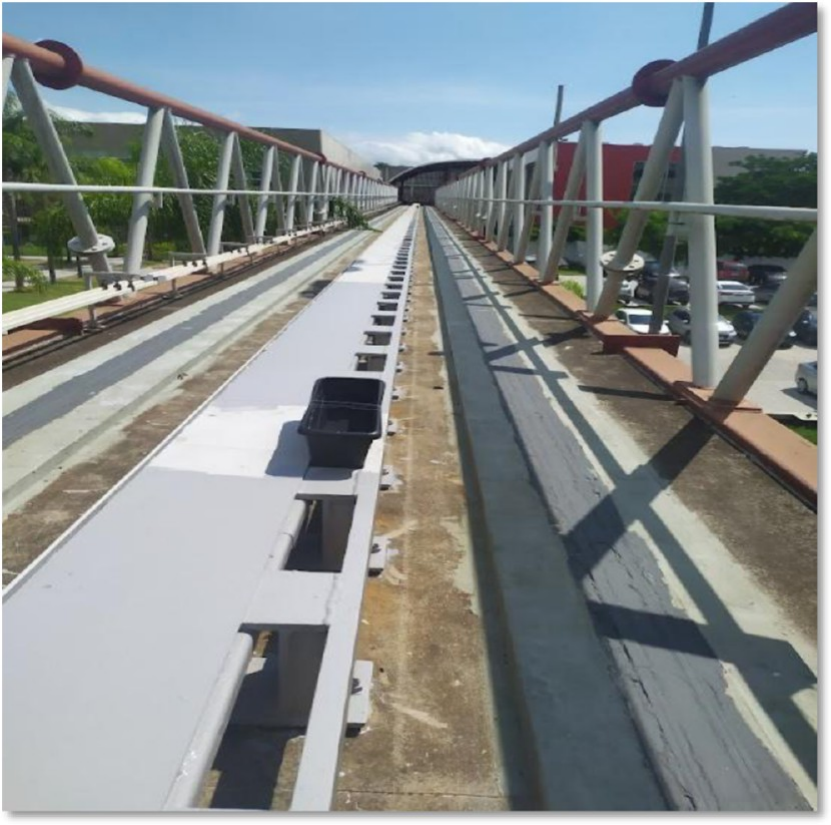
Plastic container for collecting rainwater along the track.

In addition to determining the electrolyte composition, pH measurements were performed using a pH meter (Kasvi), and the conductivity was measured with a conductivity meter (Bel) immediately after sample collection.

Routine inspections were conducted to map the extent of the corrosion process and identify the key parameters contributing to rail degradation. Initially, the rails were photographed while still fixed to the platform to document damage on the surface directly exposed to the atmosphere. Subsequently, a 1 m section of the track was disassembled and photographed to assess the extent of damage in the buried portion of the rail.

### 2.2 Immersion Tests

Immersion tests were conducted in a synthetic rainwater solution to estimate representative corrosion rates and morphology, using a composition adapted from Hernandez et al.^13^ due to the similarity with the composition of the sample collected in the Assessment of Operational Conditions, with the pH adjusted to 5.42 (the same pH of the sample collected on the rails.)

To avoid damage to the electronic equipment used during electrochemical tests, it was necessary to use the NdFeB magnets in a demagnetized state.

Demagnetized test specimens were embedded in epoxy resin and sequentially ground with 100, 220, 320, 600, and 1200 grit sandpaper and then polished with alumina until a mirror-like finish was achieved. The samples in contact with the electrolyte were exposed to a system simulating sunlight exposure, maintaining an approximate temperature of 40 °C.

After immersion times of 5, 10, 15, 30, 45, and 60 days, the specimens were analyzed by Scanning Electron Microscopy (SEM), in a Tescan microscope model Vega3, and Energy Dispersive X-ray Spectroscopy (EDS), using a Detector X-Flash 630 M of Bruker Nano GmbH. The corrosion products were further analyzed via X-ray diffraction (XRD), which were performed on a benchtop diffractometer (Rigaku MiniFlex II) using Cu Kα radiation, with diffraction angles ranging from 10° to 90°.

### 2.3 Electrochemical Test

NdFeB samples were subjected to polarization tests to investigate the corrosion process, conducted in synthetic rainwater and 3.5% w/v NaCl solution, a commonly used electrolyte in the literature for immersion tests.^14–16^

A Potentiodynamic Polarization test was performed in a three-electrode system using a platinum counter electrode and a saturated calomel reference electrode. The electrochemical tests were carried out using an Autolab potentiostat (Metrohm), and the data were acquired and analyzed with Nova 2.15 software. The potential variation ranged from −0.2 to 0.2 V relative to the open circuit potential (OCP), with a 1 mV·s^–1^ scan rate. The time of the OCP measurements was 60 s. The corrosion potential (*E*_corr_) and the corrosion current *I*_corr_ were determined by the Tafel’s extrapolation method.

## 3 Results and Discussion

### 3.1 Visual Inspection

The initial images obtained along the track (Figure 2) indicate that the corrosion process has progressed aggressively, causing significant structural degradation. The rail surface is not uniform, with regions exhibiting substantial material loss.

**Figure 2:**
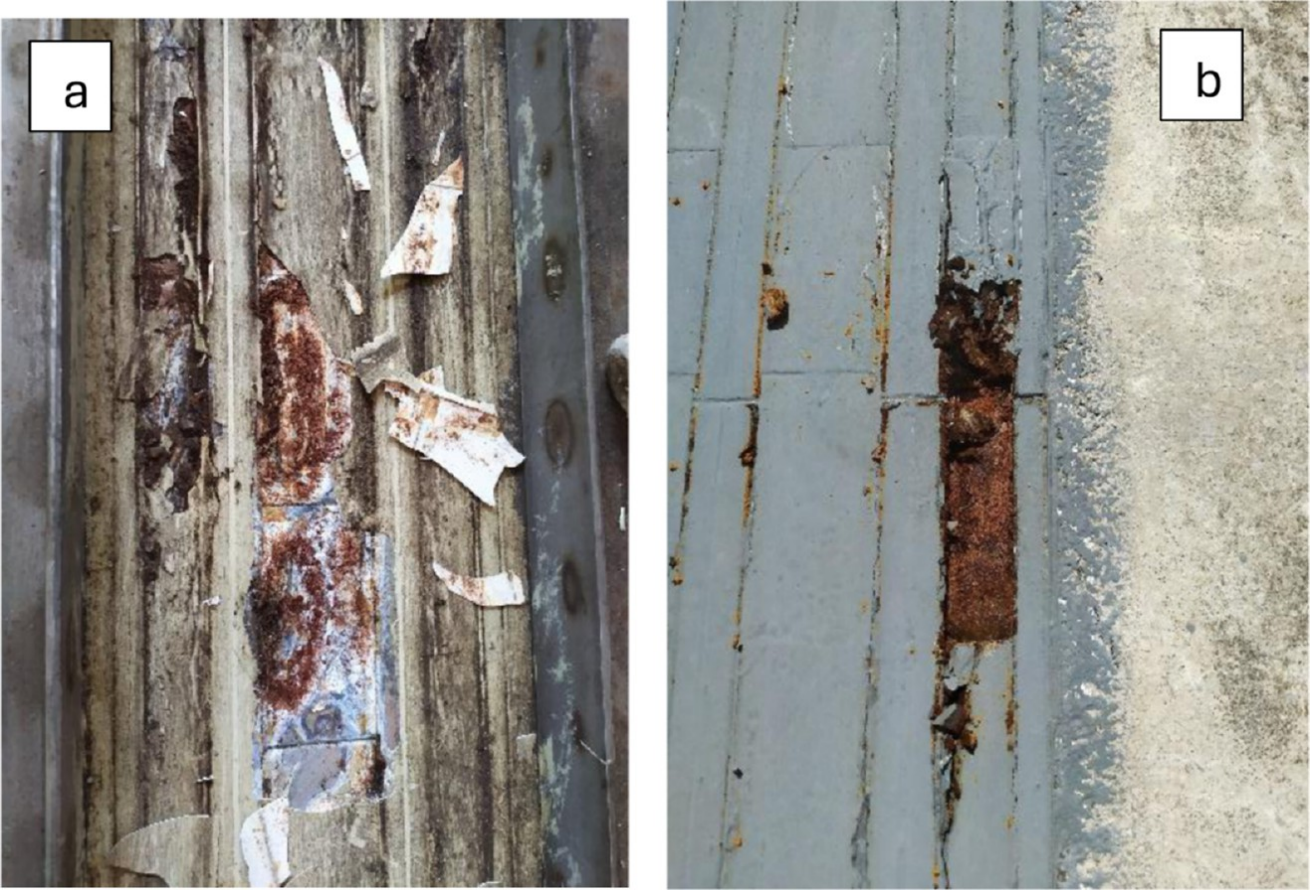
Evolution of the corrosion process on the MagLev rails after the application of epoxy coatings without the use of a blanket: (a) paint with niobium pentoxide as anticorrosive pigment and (b) paint rich in zinc.

Furthermore, corrosion mitigation was not achieved, despite the application of protective coatings. In the coated regions, delamination of the protective layer and the presence of corrosion products are evident, highlighting the continued advancement of the degradation process.

To enhance the protective barrier provided by the coating, a waterproofing membrane was applied below the zinc-rich paint. However, as shown in Figure 3, the corrosion process proved to be even more aggressive, leading to structural expansion and fragmentation of the metal.

**Figure 3:**
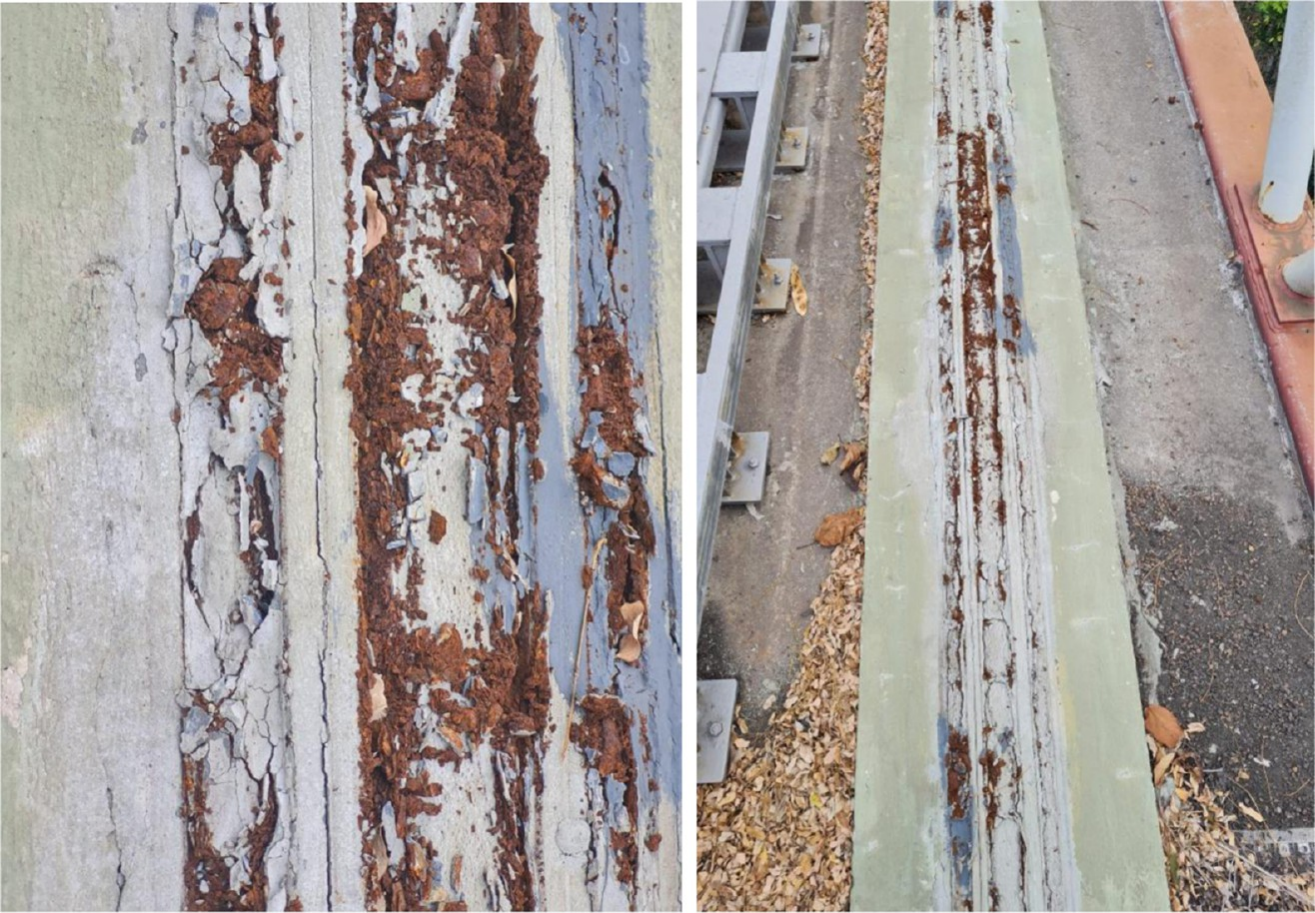
Progress of the MagLev corrosion process after application of the painting scheme using zinc-rich paint and blanket.

According to the technicians responsible for track maintenance, the onset of the first signs of degradation beneath the paint layer containing niobium pentoxide occurred after approximately 8 months, whereas for systems using zinc-rich paint, signs of failure were observed after approximately 6 months.

As the images were analyzed, evidence suggested that an increased impermeability significantly accelerated the corrosion process. This outcome contrasts with the expected effect of improving the barrier efficiency, which should ideally enhance corrosion resistance.

Yang et al. have demonstrated the benefits of applying coatings to the surface of NdFeB magnets to improve corrosion resistance. In the work, an epoxy coating with Zn/Al particles was applied, and the coated samples exhibited corrosion only after 936 h of salt spray exposure in a NaCl-rich atmosphere. In contrast, uncoated samples developed corrosion products within 24 h of immersion.^17^

Observing the previous results, conducting a more in-depth investigation of the entire rail structure became necessary rather than focusing solely on the surface. Consequently, a section of the track structure was dismantled for further analysis.

As seen in Figure 4, the corrosion process on the lower part of the rail was even more extensive, with the magnet’s surface pulverized and a significant amount of fine disaggregated particles present.

**Figure 4:**
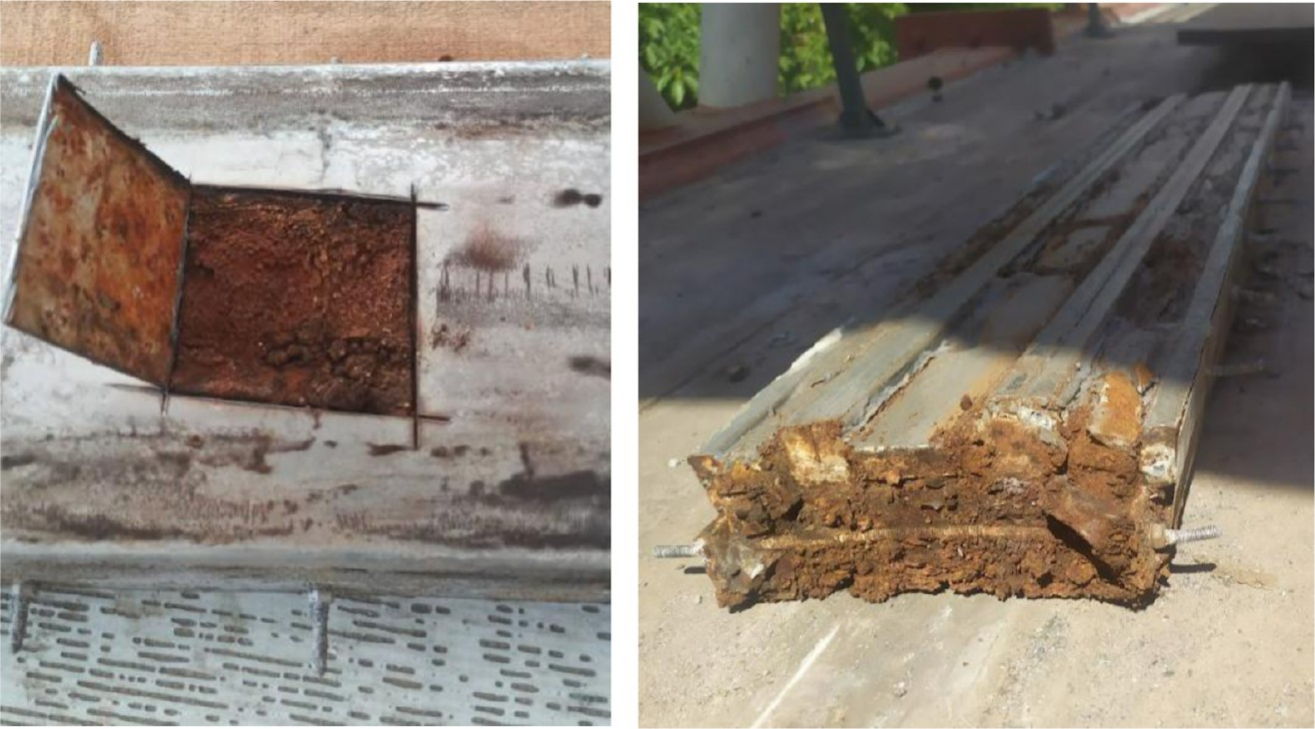
Bottom and side view of the disassembled rails.

Table 1 presents the average values of magnetic field measurements for both the most severely degraded sections of the track and recently installed rails (with no apparent corrosion). The results indicate a 58% reduction in magnetic properties accompanied by the loss of solid material—an expressive decrease when compared to the findings of Mello (2022), who had previously reported a 25% loss in magnetic field intensity in areas of the MagLev track where visible surface oxidation was already present.^12^ Isotahdon et al. presented results that corroborate these findings, concluding that part of the magnetic properties of their samples was lost due to mass loss. However, their evaluations indicated that the reduction in magnetic flux exceeded the loss of solid material, suggesting that material detachment alone is not solely responsible for the decrease in magnetic properties.^18^

**Table 1:** Magnetic Field Measurements

	Blank	Region of Most Severe Degradation
Magnetic iField (gauss)	–7087,84	–2970,40
% Loss of Magnetic Properties	-	58

According to Zhang et al., 10% of the observed magnetic property losses in their analyses was attributed to modifications in the microstructure of the magnets due to oxidation processes,^8^ which could be considered a secondary effect of corrosion progression.

When analyzing the assembly of the MagLev rail structure,^11^ it is evident that it combines three different materials: carbon steel, stainless steel, and NdFeB magnets. It is well-known that these materials have distinct electrochemical potentials and that the contact of materials with different potentials in an electrolyte leads to galvanic corrosion, accelerating the material degradation.^14,19–24^

Thus, this is believed to be one of the reasons for the advanced degradation of the structure as it presents multiple anodic and cathodic regions. In addition to the combination of different materials, it was observed that the structure used to contain the magnets has a design that facilitates water accumulation in its lower region. This accumulation creates an ideal environment for the corrosion process, considering that electrochemical reactions require an anodic and cathodic region, electrical contact, and an electrolyte. In this case, the electrolyte is presumed to be rainwater. This hypothesis is further supported by the previous observation that the lower part of the rail exhibited a significantly more severe corrosion.

During the evaluations, it was also observed that applying an epoxy coating solely on the upper face of the rail seemingly increased the corrosion rate. In this specific case, it is believed that the coating acted as an inverted barrier, trapping water vapor generated in the lower part of the structure, delaying its release, and creating an even more aggressive environment. This behavior resembles that observed in filiform corrosion processes, which are characterized by occurring in humid environments with organic coatings, where the corrosion propagates along the interface between the coating and the metallic substrate due to the presence of an electrolyte in this region.^25^ However, unlike typical filiform corrosion—in which moisture penetrates through coating defects to reach the substrate—it is believed that in this case the primary contributor to the accumulation of water at the interface is the lower part of the structure, as previously described.

### 3.2 Exposure Conditions

Temperature measurements along the rail revealed that the lowest temperatures occurred early in the morning, with a minimum of 10.3 °C. In contrast, the highest temperatures recorded on the magnets were observed between 12:00 and 1:00 p.m., when solar radiation was at its peak, reaching up to 53.5 °C.

According to the literature the corrosion process is directly influenced by environmental temperature, that it is able to accelerate the kinetics of the reaction,^26^ making it a critical parameter in this investigation. Considering that the highest temperatures recorded in Rio de Janeiro—where the study object is located—during the analysis period approached 40 °C, this temperature was selected for the immersion tests.

Determining the electrolyte composition to which the magnets are exposed is a crucial step in this study, as it allows for a more accurate reproduction of the operational conditions at a laboratory scale. The characterization of rainwater collected from the MagLev Cobra platform is presented in Table 2, showing that the rails are exposed to an acidic environment with a conductivity of approximately 8.5 μS.cm^–1^. Given the low ionic concentration of the collected samples and their similarity to those described by Hernandez et al. for the city of Rio de Janeiro, the composition reported by these authors was used for the electrochemical and immersion tests, with only pH adjustment performed using a 0.1 mol·L^–1^ HCl solution.^27^

**Table 2:** Rainwater Composition.

Ions	Na^+^	NH_4_^+^	Ca^2+^	Mg^2+^	K^+^	Cl^–^	NO_3_^–^	SO_4_^2–^	NO_2_^–^
Concentration (ppm) – Rainwater	0.65	0.163	1.59	0.145	0.158	0.098	0.34	0.015	0.01
pH		5.42							
Conductivity (μS·cm^–1^)		8.5							

#### 3.2.1 Electrochemical Polarization Tests

Electrochemical polarization was conducted to investigate the NdFeB magnet’s corrosion when exposed to synthetic rainwater. For comparison purposes, the test was also performed in a 3.5% m/m NaCl solution (Chart 1).

**Chart 1:**
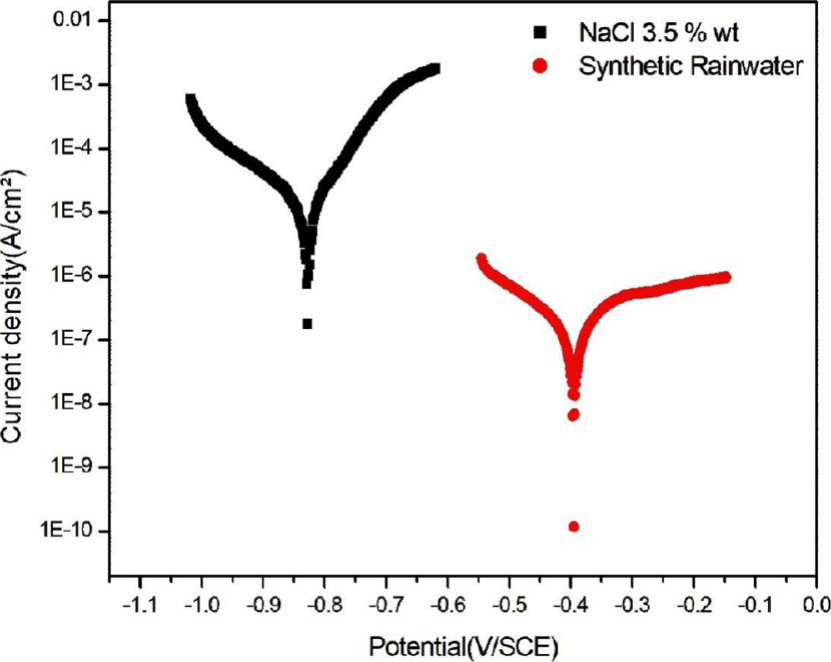
Electrochemical polarization of magnet samples in synthetic rainwater and in 3.5% m/m NaCl.

In Chart 1, it can be observed that when exposed to NaCl, the magnet exhibits a greater tendency toward corrosion than the sample immersed in synthetic rainwater. This assertion is based on the fact that the curve corresponding to rainwater is shifted further to the right, indicating a corrosion potential (*E*_corr_) of −395 mV. In contrast, in the NaCl test, the magnet presents an *E*_corr_ of −828 mV. The *E*_corr_ parameter is associated with the material’s susceptibility to undergo oxidative processes; thus, the higher this parameter, the more resistant the material is to electron loss. Conversely, the corrosion current density (*I*_corr_) is often used to describe the evolution of oxygen reduction in the medium alongside the anodic dissolution reaction of the metallic material.^28^ These parameters are obtained using the Tafel extrapolation method. The lower the *I*_corr_ value, the lower the electron flux in the system, suggesting a lower corrosion rate of the material. The parameters obtained through the Tafel method are presented in Table 3.

**Table 3:** Tafel Parameters Obtained by Electrochemical Polarization

Electrolyte	*E*_corr_ (mV)	*I*_corr_ (A)
Rainwater	–395	1.1 × 10^–7^
NaCl 3.5%_w/w_	–828	1.1 × 10^–5^

Another important aspect to be observed in the rainwater curve is the appearance of a plateau on the right side corresponding to the anodic reactions. This plateau indicates that despite a variation in potential within the system there is no significant increase in current flow through the material, suggesting a passivation process on the metal surface. During passivation, the oxide generated by the metal oxidation process forms a protective layer capable of shielding the material’s surface from the progression of the corrosion phenomenon. At the end of the test, the formation of a golden film on the magnet can be observed, further supporting the hypothesis of a protective layer formation on the alloy.

The passivation phenomenon is not observed for the specimen immersed in NaCl solution, this divergence can be explained by the high chloride concentration in the NaCl sample, which, according to Chitrada et al., leads to an increase in pitting corrosion, hindering the formation of the passive layer and lowering the corrosion potential of the sample, as can also be observed in Chart 1.^29^

### 3.3 Corrosion Morphology

In Figure 5a, small cracks appear on the material’s surface after 5 days of immersion, along with some vacancies in certain regions. This surface morphology suggests a combination of two corrosion processes: pitting and cracking of the metallic structure. According to the literature, the initial appearance of cracks recurrently occurs in the neodymium-rich phase of this specific type of material,^8^ this can be observed in the images obtained through Energy Dispersive X-ray Spectroscopy (EDS) analysis of the sample after 5 days of immersion (Figure 6), where a higher concentration of Nd is detected precisely in the cracking region.

**Figure 5:**
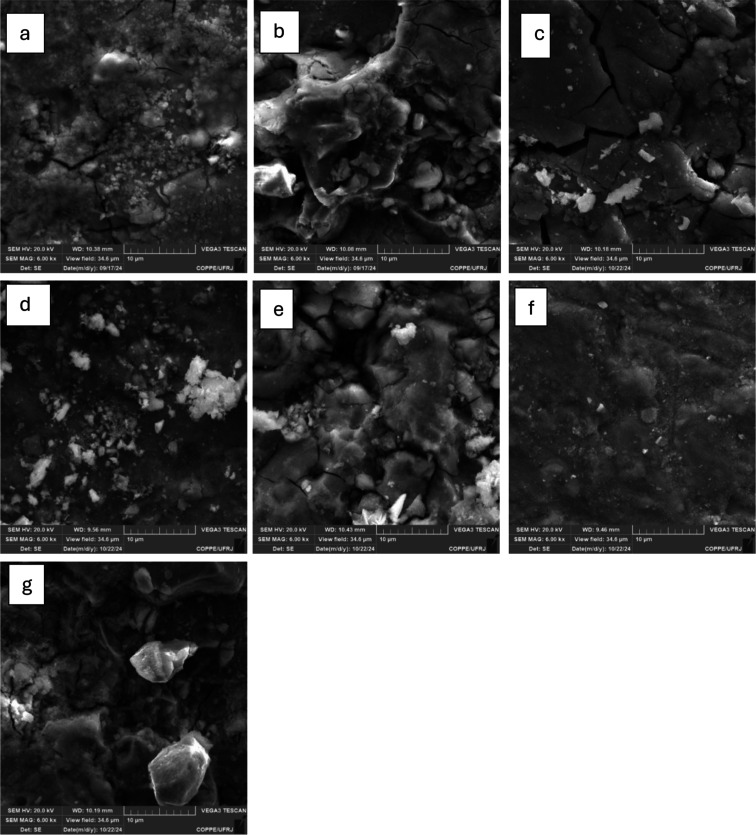
Images obtained by SEM of the samples after the immersion test: a, b, c, d, e, f, and g are 5, 10, 15, 20, 30, 45, and 60 days, respectively.

**Figure 6:**
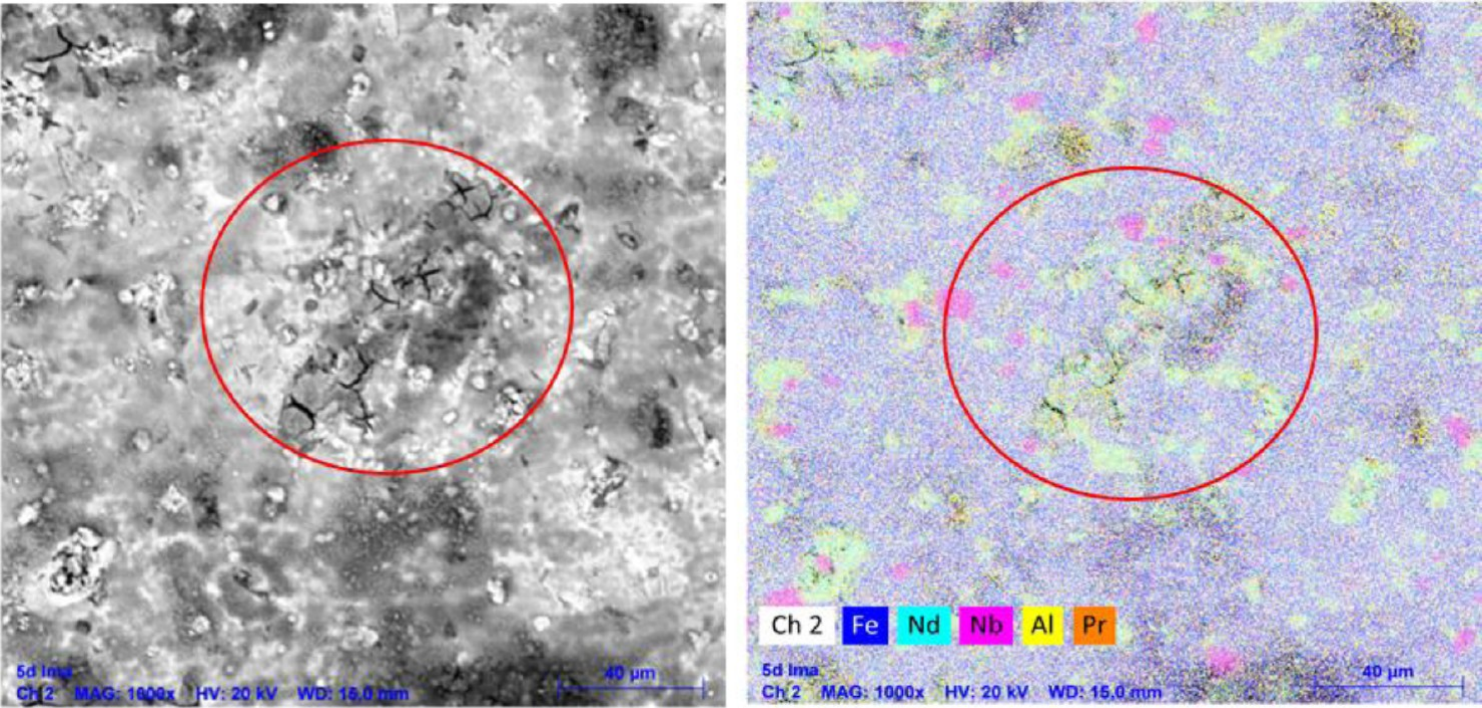
EDS of the sample after 5 days of immersion.

As the immersion time progresses, material detachment is observed (Figure 5b), indicating that corrosion has advanced to initiating phase separation and the formation of more profound and more pronounced valleys on the surface. After 15 days of immersion, the material’s structure shows wider cracks compared to those observed at the beginning of the test, which may result from the progression of pitting corrosion, leading to increased fragility of the intergranular structure.

At 20 and 30 days of immersion, the sample’s surface exhibits a more irregular structure, possibly due to the formation of a corrosion product layer, i.e., the progression of the material’s passivation process. This process is confirmed in Figure 5d, where a more uniform surface topography is observed, indicating the completion of protective film formation.

After an additional 15 days of immersion, the material presents an entirely irregular surface, suggesting significant detachment of particulate material; this indicates that the peak of surface oxidation occurs at 45 days with the formation of an oxidized material layer, which subsequently begins to degrade over the following 15 days.

The macroscale development of this film can be observed in Figure 7. The initial formation of an irregular film is visible over the first 30 days of immersion, which becomes nearly uniform after 45 days but detaches entirely after 60 days of testing. This result suggests that the protective layer formed is weakly adherent, providing only limited protection to the metallic substrate despite being a passivation phenomenon.

**Figure 7:**
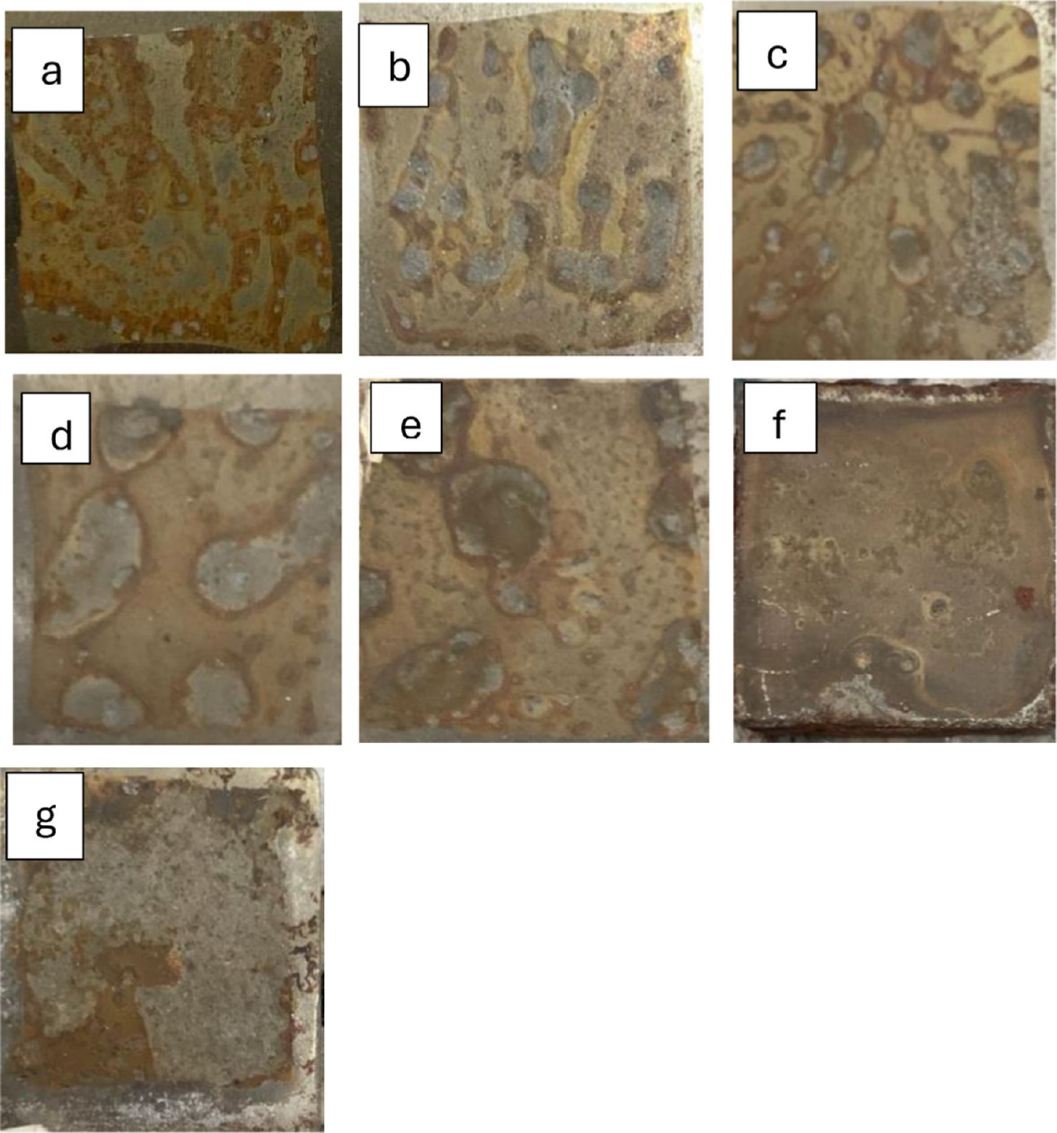
Photographs in macroscale of the specimens after the immersion test: a, b, c, d, e, f, and g are 5, 10, 15, 20, 30, 45, and 60 days, respectively.

Another result that can be derived from the EDS analysis images concerns the high concentration of iron on the sample surface, indicating that the NdFeB alloy is predominantly ferrous. In Table 4, the elemental composition of the magnet sample is presented, where it can be observed that neodymium is the second most abundant metal.

**Table 4:** Elemental Analysis Obtained by EDS

Element	%mass	%atomic
Iron (Fe)	52,74	80,75
Neodymium (Nd)	21,51	12,75
Niobium (Nb)	1,17	1,07
Aluminum (Al)	0,67	2,11
Praseodymium (Pr)	5,46	3,31

### 3.4 XRD

In Figure 8, it is possible to observe that the corrosion product analyzed by XRD is predominantly composed of variations of iron-rich corrosion compounds. The diffraction peaks identified at 2θ = 27.047°, 36.296°, 46.914°, 52.714°, and 60.686° correspond to the formation of lepidocrocite, according to PDF 08-0098. Additionally, the peaks at 14.88°, 36.373°, 38.049°, and 46.865° are attributed to iron hydroxide (Fe­(OH)_3_), in agreement with PDF 38-0032. Regarding the presence of Nd in the corrosion product, the peaks at 34.27°, 43.38°, and 68.76° indicate the presence of neodymium hydroxide (Nd­(OH)_3_), according to the PDF 13-0085. The absence of peaks associated with B-rich phases raises the hypothesis that this presence in the corrosion products is negligible compared to iron and neodymium. Therefore, it can be inferred that the phases disaggregated during the immersion test, as observed in the SEM results, are the iron-rich phase and the neodymium-rich phase.

**Figure 8:**
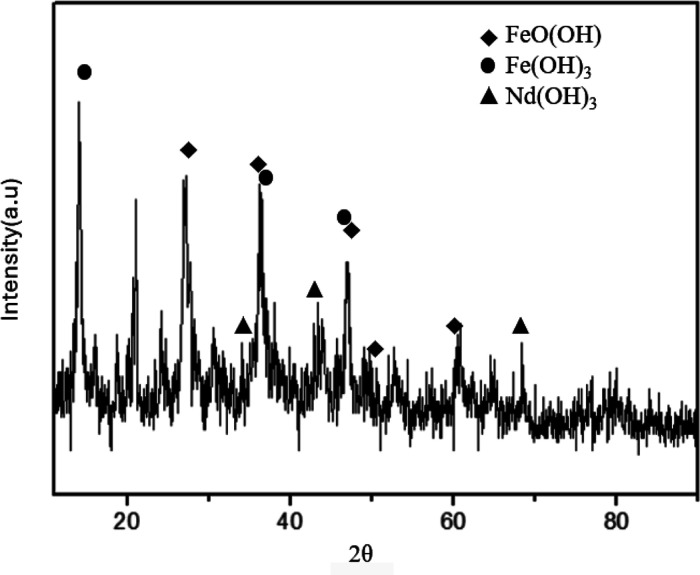
XRD of corrosion products.

## 4 Conclusions

Based on the results obtained during field inspections, it was observed that, compared to the top of the rail structure, the lower part—sheltered by the metallic channel—exhibited a significantly more severe degradation process, including the disintegration of solid phases. As the study progressed, it was found that the channel enclosing the magnets in the system is a continuous structure, which hinders the drainage of the electrolyte, thereby increasing the retention time within the structure. The present study highlights the necessity of incorporating a drainage system into the MagLev tracks to minimize electrolyte accumulation at the lower part of the system, which significantly contributes to the advanced corrosion process observed in the magnets embedded within the tracks. Considering that the tracks are exposed on an uncovered platform and that painting only one face of the track accelerated the corrosion process by trapping vaporized electrolytes within the channel for a prolonged period, it was concluded that another effective strategy to extend the service life of the tracks would be to coat all faces of the magnets with a protective paint scheme. This additional coating would provide an extra layer of corrosion resistance beyond the original factory-applied protection.

Regarding the corrosion process, it was observed that over time, corrosion progresses, leading to the gradual formation of a more uniform oxide layer on the alloy surface. After 45 days, this protective barrier ruptures, restarting the corrosion process in the underlying layer. Furthermore, the corrosion mechanism initiates with the formation of microstructural cracks in the magnet, which subsequently develop into pitting corrosion, followed by detachment of the solid material. This phenomenon explains the pulverization of metallic materials in areas with more advanced corrosion.

The polarization test results corroborate those obtained in the immersion test, as the appearance of a plateau on the anodic side of the polarization curve suggests a passivation process; this indicates the formation of a protective oxide layer on the material’s surface, which plays a crucial role in temporarily mitigating corrosion progression.
